# Treatment of Hæmaturia

**Published:** 1842-12-17

**Authors:** 


*Treatment of Hæmaturia.*—In some cases of hæmaturia, the bladder is completely filled with coagulated blood, and the surgeon finds the greatest difficulty in relieving the organ of its contents.

In five cases of an extreme and urgent kind, M. Leroy d'Etiolles succeeded in evacuating the bladder by frequently introducing a very large gum elastic bougie, without a stilette. In one case the instrument was passed more than 100 times in a few hours, and two quarts of coagulated blood removed.—***Prov. Med. Jour.***, Sept. 17, 1842.

